# Design of Novel Functional Conductive Structures and Preparation of High-Hole-Mobility Polymer Transistors by Green Synthesis Using Acceptor–Donor–Acceptor Strategies

**DOI:** 10.3390/polym16030396

**Published:** 2024-01-31

**Authors:** Shiwei Ren, Sichun Wang, Jinyang Chen, Zhengran Yi

**Affiliations:** 1 Advanced Materials Platform Laboratory, Zhuhai Fudan Innovation and Science Research Center, Guangdong-Macao In-Depth Cooperation Zone in Hengqin 519000, China; shiwei_ren@fudan.edu.cn; 2 Laboratory of Molecular Materials and Devices, State Key Laboratory of Molecular Engineering of Polymers, Department of Materials Science, Fudan University, Shanghai 200438, China; sichunwang@fudan.edu.cn; 3 Alternative Technologies for Fine Chemicals Process of Zhejiang Key Laboratory, Shaoxing University, Shaoxing 312000, China

**Keywords:** novel acceptor, conductivity, Suzuki polycondensation, planarity, high mobility, semiconductor

## Abstract

The design of novel acceptor molecular structures based on classical building blocks is regarded as one of the efficient ways to explore the application of organic conjugated materials in conductivity and electronics. Here, a novel acceptor moiety, thiophene-vinyl-diketopyrrolopyrrole (TVDPP), was envisioned and prepared with a longer conjugation length and a more rigid structure than thiophene-diketopyrrolopyrrole (TDPP). The brominated TVDPP can be sequentially bonded to trimethyltin-containing benzo[c][1,2,5]thiadiazole units via Suzuki polycondensation to efficiently prepare the polymer PTVDPP-BSz, which features high molecular weight and excellent thermal stability. The polymerization process takes only 24 h and eliminates the need for chlorinated organic solvents or toxic tin-based reagents. Density functional theory (DFT) simulations and film morphology analyses verify the planarity and high crystallinity of the material, respectively, which facilitates the achievement of high carrier mobility. Conductivity measurements of the polymeric material in the organic transistor device show a hole mobility of 0.34 cm^2^ V^−1^ s^−1^, which illustrates its potential for functionalized semiconductor applications.

## 1. Introduction

**Figure 1:**
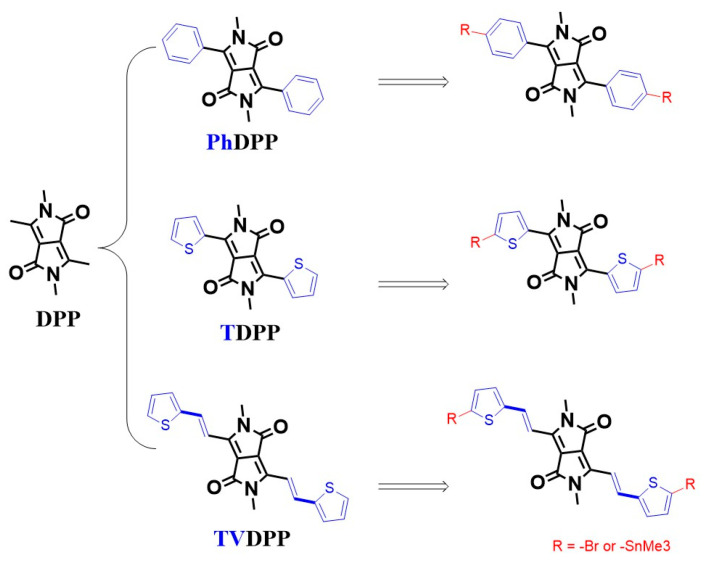
Common binding modes of DPP to aromatic rings and the molecular structure of TVDPP.

Carrier transport in conjugated organics based on alternating arrangements of single and double bonds enables conductive applications, which is the most fundamental chemical logic of organic optoelectronic research [1,2,3]. Various electronic applications including organic light-emitting transistors, chemical sensors, electrochemical transistors, thermoelectrics, perovskite solar cells, etc., are composed of organic small molecules and/or polymers as the core determinants of device performance [4,5,6,7,8,9,10]. Typical studies of macromolecular semiconductor materials have been carried out in relation to their good chemical modifiability and functionalization capabilities, as well as to their good film-forming properties, which distinguish them from small molecules [11,12,13]. Compared to the donor–donor type or acceptor–acceptor type polymers, the donor–acceptor type materials possess better modulation of the frontier molecular orbital (FMO) levels and thus are promising potential materials for device-oriented applications [14,15,16]. The FMO energy levels include the highest occupied molecular orbital (HOMO) and the lowest unoccupied molecular orbital (LUMO) levels, which are dominated by the type and content of donor and acceptor in the molecular architecture, respectively [17,18]. Suitable acceptor motifs tend to contain strong electron-withdrawing groups, typically represented by diketopyrrolopyrrole, which is always abbreviated as DPP [19,20]. DPP cannot be directly polymerized with the donor but requires the introduction of aromatic substituents (e.g., thienyl (T-) or phenyl (Ph-) rings) for Pd-catalyzed Stille coupling or Suzuki coupling reactions (Figure 1) [21,22]. Thelakkat et al. reported that polymers prepared from alternating arrangements of TDPP and monothiophene showed a moderate mobility of 0.09 cm^2^ V^−1^ s^−1^. Further, by inserting the fluorine atom with strong electron-withdrawing properties into the thiophene ring, the material transitions from P-type migration to ambipolar migration [23]. The introduction of more thiophene units facilitates the enhancement of the donor properties of the material. Li et al. prepared a material of PTDPP-2T and showed a high hole mobility of 0.80 cm^2^ V^−1^ s^−1^ [24]. Replacement of the bis-thiophene with a fused thieno [3,2-b]thiophene moiety with better planarity led to a further improvement in device performance. Sona et al. prepared PTDPP-TT materials and revealed an average mobility of 0.90 cm^2^ V^−1^ s^−1^ [25]. These structural analogs are also used in solution-processed integrated electronic components due to their excellent solubility and show a mobility of 0.1 cm^2^ V^−1^ s^−1^ and low hysteresis [26]. The introduction of a double bond between thiophene and thiophene facilitates the formation of conformational locks, thus further ensuring the coplanarity of the material. Kim et al. reported polymers based on PTDPP-TVT and showed a hole mobility close to 1.50 cm^2^ V^−1^ s^−1^ [27]. Thus, the important role of intramolecular conformational stabilization and structural ordering in the enhancement of conductivity through chemical bonding can be seen. In the case of TDPP, the thiophene ring is connected to the DPP unit by a single bond, and the rotatability of its orientation may lead to a decrease in the overall planarity of the material. Nielsen et al. suggest that the weak force formed by the S atom on the thiophene ring with the O atom on the carbonyl group reduces the dihedral angle of the whole molecule and enhances its coplanarity [28]. However, the possible repulsive forces from the H atoms of thiophene and the H atoms on the side chains cannot be ignored. Wang et al. suggest that the thiophene orientation is more inclined to the opposite direction. The non-covalent bonding interactions employed to stabilize the configuration are mainly intramolecular hydrogen bonding O…H and S…H interactions formed by S atoms with H atoms on alkyl chains [29]. Crystal analysis shows that the orientation of the thiophene ring is partly associated with the choice of alkyl chains and growth conditions, which undoubtedly results in the presence of regioisomers and decreases the degree of the long-range ordered structure of the polymer [30]. Well-formed planarity facilitates the formation of good stacking between chains, as well as increasing the effective conjugate length, thus improving the charge mobility of the device [31]. One of the solutions to resolve isomers due to asymmetric structural rotation is the introduction of symmetry groups to prepare units such as the PhDPP. However, the same significant decrease in planarity caused by the repulsive interaction between the H atoms is present on the benzene ring and the side chain [32,33]. Kagan et al. reported a hole mobility of only 0.04 cm^2^ V^−1^ s^−1^ for the polymer PPhDPP-2T [34].

Here, we propose another solution to lock the conformation of the molecule with the insertion of a bridging group of the olefin. The structure of TVDPP is supposed to be extremely planar while providing a satisfactory conjugate length [35]. Further, for the purpose of reducing the hazardous effects of tin-containing reagents on humans and the environment, the TVDPP monomer was polycondensation with benzothiadiazole (BSz) via the Suzuki condition to prepare materials with acceptor–donor–acceptor structures [36,37,38]. The conductivity of PTVDPP-BSz was tested in a polymer field effect transistor (PFET).

## 2. Materials and Methods

Reaction precursors such as pyridinium 4-toluenesulfonate (PPTS), sodium hydroxide (NaOH), triphenylphosphine (PPh_3_), etc., and common solvents such as tetrahydrofuran (THF), ethyl acetate (EtAc), petroleum ether (PE), dichloromethane (DCM), etc., were acquired from Merck (Darmstadt, German) and Alfa Aesar (Haverhill, UK) and used as received. Reaction precursor 2,1,3-benzothiadiazole-4,7-bis(boronic acid pinacol ester) (BSz) and tetra(triphenylphosphine)palladium [Pd(PPh_3_)_4_] were purchased from SunaTech (Suzhou, China) and used directly. The preparation of intermediates and final product PTVDPP-BSz was described as follows.

### 2.1. Synthesis Procedures for Small-Molecule Intermediates and Monomer

Intermediate B: To a solution mixture of 2-octyldodecylamine (R-NH_2_, 7.44 g, 25 mmol) in 100 mL of THF, argon was passed and stirred at 0 °C. Triethylamine (Et_3_N, 2.02 g, 20 mmol) was then fed into the flask. Trans-butenedioyl chloride (compound A, 1.53 g, 10 mmol) was pre-dissolved in THF (10 mL) and fed dropwise into the mixture. The reaction was completed by removing the ice bath and stirring the mixture for 2 h at room temperature. After removal of the solvent by distillation under reduced pressure, the mixture was extracted with ethyl acetate. The organic phases were collected and combined, then washed with saturated brine. The organic phase was dried over anhydrous sodium sulfate (Na_2_SO_4_) and then concentrated before separation by column chromatography (EtAc/PE = 1:4) to give a greyish-white powder of 5.06 g in 75% yield. Mass: Calculated for [C_44_H_87_N_2_O_2_]^+^: 675.6768; Found: 675.6767. The ^1^H NMR and ^13^C NMR profiles and characterization of compound B are shown in Figures S1 and S2 in Supplementary Materials, respectively.

Intermediate C: To a mixture of isopropenyl acetate (50 mL) of intermediate B (4.44 g, 6.58 mmol), p-toluenesulfonic acid (p-TsOH, 7.0 mL, pure, 12% in acetic acid) was added. The system was kept at 95 °C for 5 h under stirring conditions. The crude was washed with hydrochloric acid (5%) and subsequently washed with saturated sodium bicarbonate solution. The organic phase was extracted using ethyl acetate, concentrated and purified by column chromatography (EtAc/PE = 1:5) to give a brown oil of 3.29 g in 66% yield. Mass: Calculated for [C_48_H_91_N_2_O_4_]^+^: 759.6979; Found: 759.6972. The ^1^H NMR and ^13^C NMR profiles and characterization of compound C are shown in Figures S3 and S4, respectively.

Intermediate D: To a solution mixture of THF and acetonitrile (3:1 of 40 mL) of intermediate C (1.52 g, 2.0 mmol) and PPh_3_ (629.5 mg, 2.4 mmol), PPTS (251.3 mg, 1.0 mmol) was added. The reaction was carried out in a thick-walled pressure-resistant flask at 100 °C for 24 h. The crude was extracted by using dichloromethane, concentrated and purified by column chromatography (EtAc/PE = 1:4) to give a yellow oil of 0.58 g in 40% yield. Mass: Calculated for [C_48_H_89_N_2_O_2_]^+^: 725.6924; Found: 725.6917. ^1^H NMR and ^13^C NMR profiles and characterization of compound D are shown in Figures S5 and S6, respectively.

Monomer TVDPP: To a mixture of toluene and methanol (5:1 of 6 mL) of intermediate D (724.7 mg, 1.0 mmol), 5-bromothiophene-2-carbaldehyde (474.7 mg, 2.5 mmol) was added, followed by L-proline (23.1 mg, 0.2 mmol) and Et_3_N (0.40 g, 4.0 mmol). The mixture was reacted under argon protection at room temperature for 12 h. The crude product was extracted with dichloromethane and then dried over anhydrous Na_2_SO_4_. The organic phase was stirred to silica gel powder and purified by column chromatography (DCM/PE = 3:2) to give a brown solid of 458.5 mg in 43% yield. Mass: Calculated for [C_58_H_91_Br_2_N_2_O_2_S_2_]^+^: 1071.4864; Found: 1071.4874. ^1^H NMR and ^13^C NMR profiles and characterization of compound TVDPP are shown in Figures S7 and S8, respectively.

### 2.2. Synthesis Procedure for Conductive Polymers PTVDPP-BSz

Polymer PTVDPP-BSz: TVDPP (100.00 mg, 93.41 µmol), BSz (36.26 mg, 93.41 µmol) and [Pd(PPh_3_)_4_] (1.10 mg, 0.93 µmol, 1%) were fed into a pre-dried 50 mL of Schlenk tube and subsequently passed through argon for ten minutes to sufficiently remove oxygen from the system. A mixture consisting of 1.0 mL of 2 M NaOH solution and 7.0 mL of toluene was inserted into the mixture through a syringe. Stirring was started, after which the solid was gradually dissolved. Argon was withdrawn after the temperature had risen to 90 °C. The reaction was terminated after 24 h, and the solution appeared dark green in color. The mixture was transferred through a pipette and added drop by drop to 200 mL of methanol solution, accompanied by rapid generation and precipitation of solids. The solid was obtained by reduced pressure filtration and purified using Soxhlet extraction technique. The removal of impurities was carried out sequentially using anhydrous methanol, acetone and n-hexane solvents under reflux conditions. The final collection of the chloroform phase afforded the expected product. Reprecipitation in methanol was again utilized, and PTVDPP-BSz was obtained by drying under reduced pressure in 82% yield. The ^1^H NMR and ^13^C NMR profiles of the polymer were not obtained due to solubility issues.

### 2.3. Characterization Steps and Details of the Intermediates and Polymer

Nuclear magnetic resonance including ^1^H NMR and ^13^C NMR spectra was recorded on a 400 M spectrometer (Bruker, Karlsruhe, Germany) with deuterated chloroform (CDCl_3_) and tetramethylsilane as the internal standard. Multiplicities are denoted as s = singlet, d = doublet, t = triplet, m = multiplet, br = broad. Coupling constants (J) are denoted in Hz and chemical shifts in parts per million (ppm). Mass spectra were collected on an Autoflex III (Bruker, Daltonics Inc, Karlsruhe, Germany). The transmitted Fourier transform infrared (FTIR) spectra of the powders were collected in vacuum using an FTIR spectrometer (Perkin-Elmer Spectrum GX, Waltham, MA, USA) with KBr pellets. The molecular weight of the macromolecule PTVDPP-BSz was assessed via gel permeation chromatography (GPC) using trichlorobenzene as eluent and polystyrene as standard (150 °C, PL-GPC50, Agilent, Santa Clara, CA, USA). Elemental analysis was carried out by measuring the powder in CHN mode on an organic elemental analyzer (Perkin Elmer 2400, Waltham, MA, USA). The thermal stability of the powder of TVDPP and PTVDPP-BSz was tested by means of a thermogravimetric analyzer in a nitrogen atmosphere over a period of 50 to 800 °C with a heating rate of 10 °C per minute (TG-DTA8122, Rigaku, Tokyo, Japan). The photochemical analyses were determined using a UV-visible spectrometer (Cary5000, Agilent, Santa Clara, CA, USA). The solution phase was tested in chloroform with the polymer at a concentration of about 0.1 mg/mL. Another sample of solutions at a concentration of around 2.0 mg/mL was spin-coated onto pre-cleaned quartz plates (0.5 cm × 1.0 cm) and thermally annealed to prepare thin films for solid-phase testing. Electrochemical tests were carried out in anhydrous acetonitrile solution in an argon atmosphere with an electrolyte of 0.1 M tetrabutylammonium hexafluorophosphate. Ag/AgCl electrode, glassy carbon electrode and platinum electrode were used as reference, working and counter electrodes, respectively. A drop of 6.0 µL of the solution (2.0 mg/mL) was pipetted onto the working electrode and left to evaporate to form a dense dark green film. For calibration purposes, the internal standard of ferrocene-ferrocene (Fc/Fc^+^) was measured under identical conditions. E_HOMO_ = −4.80 − (E_ox_^onset^ − 0.40) eV; E_LUMO_ = −4.80 − (E_red_^onset^ − 0.40) eV, in which E^onset^ is the oxidation or reduction potential value relative to the Fc/Fc^+^ [39]. The scan voltage was set from −1.6 V to 2.0 V, and the first loop cycle was selected as the result. The thermally annealed film was measured by atomic force microscopy (AFM) to visualize the morphology, which was prepared under the same conditions as employed for the PFET (Nanoscope V, Bruker, Germany). Grazing incidence wide-angle X-ray scattering (GIWAXS) measurements were acquired at 14 B/15 U beamline station in Synchrotron Radiation Facility (Shanghai, China). The sample was identical to the one used for the analysis of the conductivity of the PFET.

### 2.4. Device Preparation for Measuring Conductivity of Material

The electrical properties of PTVDPP-BSz films were investigated using bottom gate/bottom contact (BG/BC) device configuration. A highly doped n-type silicon (Si) wafer with a 300 nm thick silicon dioxide (SiO_2_) layer was used as the gate electrode and dielectric layer (0.6 cm × 0.6 cm). The substrate was ultrasonically cleaned with deionized water and then soaked with a piranha solution (volume ratio of hydrogen peroxide to sulfuric acid = 1:2) for 30 min. Afterward, the substrate was ultrasonically cleaned in sequence with deionized water, ethanol and isopropanol, respectively, for 10 min, followed by drying with nitrogen. A mask version was used for the fabrication of source and drain electrodes with channel widths and lengths of 1400 µm and 30 µm, respectively. Vacuum deposited was used for the thermal evaporation of electrodes by gold (Au), and the thickness of the electrode was approximately 30 nm. The polymer PTVDPP-BSz was pre-dissolved in chlorobenzene solvent at a concentration of 5 mg/mL and kept stirring overnight on a hot table at 60 °C to sufficiently dissolve. Then, the polymer solution was deposited onto the substrate with Au electrodes at a constant spin-coating rate of 3000 rpm for 60 s under ambient conditions to form a thin film. Finally, the polymer film was annealed in an oven at 150 °C for a period of 30 min and, afterward, slowly cooled to room temperature to eliminate the corresponding solvent to complete the construction of the device.

Electrical characteristic curve data were measured using a 4200-semiconductor characterization system (Keithley, Cleveland, OH, USA) operating under ambient conditions. The saturation hole mobility values (*µ*) were obtained by the transfer characteristic curves, following the following formula:

*μ* = ($\frac{\partial \surd {|I}_{DS}|}{\partial V_{GS}}$)^2^ · $\frac{2L}{WCi}$ (*I_DS_*, *V_GS_*, W and L are the source-drain current, gate voltage, channel width and channel length, respectively; Ci is the capacitance per unit area of the 300 nm SiO_2_ of gate dielectric layer, Ci = 11.5 nF/cm^2^) [40,41].

## 3. Results

### 3.1. Preparation Route and Molecular Composition Analysis of the Polymer PTVDPP-BSz

**Figure 2:**
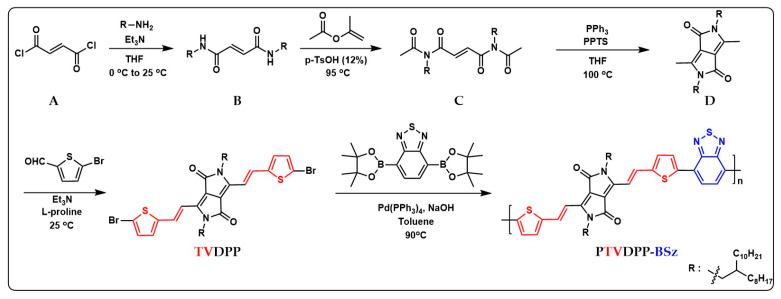
Sequential preparation of monomeric TVDPP from intermediates of A to D and synthesis of the polymer PTVDPP-BSz prepared based on the Suzuki coupling method; the red and blue parts correspond to the abbreviations of the corresponding chemical structures.

In the previous section, a comprehensive protocol for the preparation of monomer TVDPP containing dibromine from intermediates via a four-step reaction pathway was presented as shown in Figure 2 below. The C–Br bond can be conveniently broken by zero-valent palladium for coupling with the borate-containing monomer BSz. BSz is an electron-deficient group with good planarity that favors donor–acceptor interactions within the polymer chain [42]. The Suzuki reaction is safer and greener than the toxic tin-containing structural units employed in Stille coupling. At the same time, the system reduces the demanding anhydrous solvent requirements for Stille reaction conditions by using trace amounts of water to dissolve the required inorganic bases. The solvent used to prepare the polymer was toluene rather than the common chlorobenzene solvent. Chlorine-containing reagents are inherently toxic compared to toluene solvents. The process of conjugated chain extension of the polymer is highly efficient and takes only 24 h. The Soxhlet extraction of the crude significantly removes the catalysts and oligomers present in the system [43].

Elemental analyses of the final product also verified the purity of the material, as shown in Table 1. The discrepancy in the experiments was around 0.3% when compared to the theoretical values of the elements contained in the minimum-repetition unit. The molecular weight of PTVDPP-BSz was estimated by GPC chromatogram (Figure S9) which showed good dispersion (Đ). The average number of repeating units in each chain was calculated to be 22 and 48, respectively, according to the ratio of the number-average molecular weight (Mn) or the weight-average molar molecular weight (Mw) to the mass of the minimum-repeating unit. The large degree of polymerization is indicative of the extremely long π–conjugation length of PTVDPP-BSz. Figure S10 shows the thermal decomposition curve of the polymer and monomer, which are both extremely thermally stable over the temperature range from room temperature to 350 °C. The polymer loss of 10% mass is close to 360 °C, which is comparable to the decomposition temperature of most DPP-based polymer systems. On the other hand, the difference in thermal stability between PTVDPP-BSz and TVDPP is mainly seen after 500 °C, while the small molecules exhibit more residual mass between 500 and 700 °C. The FTIR spectra of the polymer and the monomer are shown in Figure S11, which exhibit a high similarity. There is a slight shift in the position of the carbonyl feature peak at 1653 cm^−1^ and 1659 cm^−1^, respectively.

### 3.2. Theoretical Simulation Calculations

**Figure 3:**
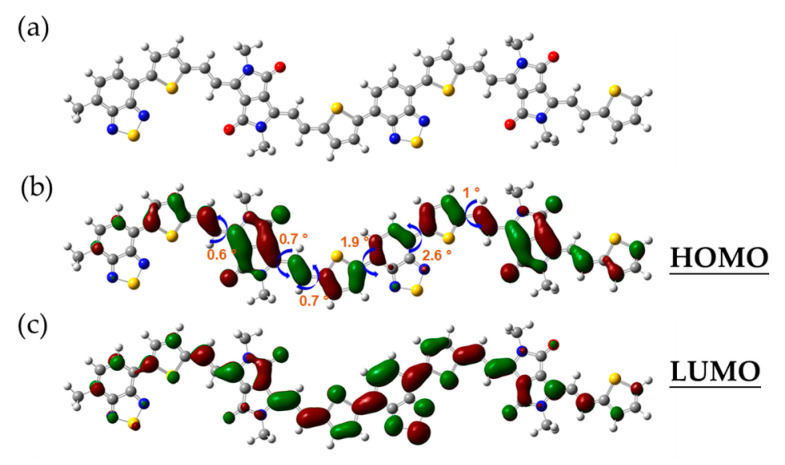
(**a**) Optimized conformation of the dimer. (**b**) Typical dihedral angle and the HOMO orbital map; (**c**) the LUMO orbital map.

To further gain insight into the internal compositional characteristics of the material of PTVDPP-BSz, a DFT-based simulation study was conducted with the B3LYP/6-31G(d) functional basis [44,45]. For the sake of simplifying the calculations, the long aliphatic chain at the nitrogen position is replaced by a methyl group, which is related to the fact that the non-conjugated components scarcely affect the FMO levels in the backbone (Figure 3a). Meanwhile, the choice of a dimer as the sample facilitates balancing the computational difficulty with accurate results, which has been accepted in various literature [46]. Typical unit-to-unit dihedral angles are shown in Figure 3b, where thiophene is connected to the DPP via an olefin as a bridge group at a dihedral angle within 1°. Bsz forms a high coplanarity with the adjacent thiophene with dihedral angles of 1.9° and 2.6°, respectively. The well-developed coplanarity promotes π−π stacking and facilitates carrier transport within and hopping between molecules, thus contributing to high-performance conductive properties. The results of Figure 3b,c elucidate the full delocalization of HOMO and LUMO within the conjugated system consisting of DPP and BSz acceptors, thiophene donors and bridging motifs. The HOMO and LUMO levels of the dimer are computed by simulation as −4.63 eV and −3.17 eV, respectively, which indicates an energy gap for 1.46 eV.

### 3.3. Photochemical Properties

**Figure 4:**
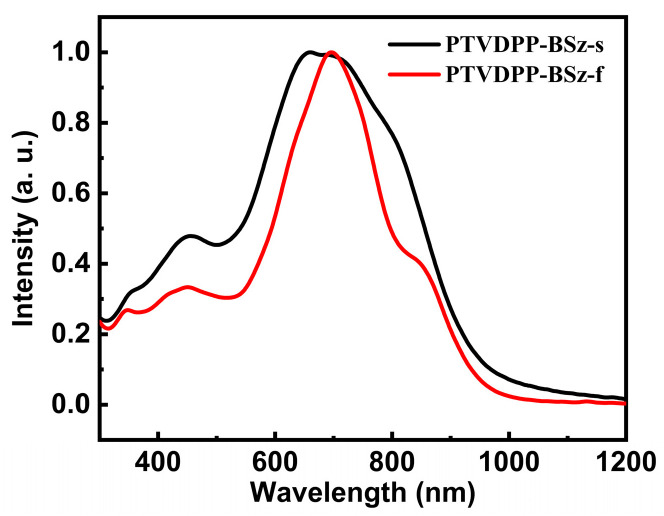
UV-Vis absorption features of polymer, with the black and red lines representing the absorption in the solution and the annealed film state, respectively.

For the study of the optical properties of polymers with long conjugated structures, we tested their ultraviolet-visible (UV-vis) absorption profiles in chloroform dilute solutions and in the solid phase of thin films. The absorption of PTVDPP-BSz is similar to that of the majority of polymers based on the donor–acceptor architecture of DPP, with dual absorption bands in both states and a broad absorption band ranging from 350 to 1100 nm (Figure 4). PTVDPP-BSz in solution displays a strong absorption band with a maximum wavelength (λ_max_) of 660 nm over the wavelength range from 500 nm to 1050 nm, which results from the intramolecular charge transfer. High energy absorption at low wavelengths occurs between 300 nm and 500 nm due to π–π* transition within the backbone. The λ_max_ of PTVDPP-BSz in thin films shows a pronounced bathochromic shift of 37 nm as compared to the λ_max_ in solution, being 697 nm. This is associated, on the one hand, with solid-state intermolecular interactions induced by the strong polarity of lactam moieties in TVDPP units, leading to a more ordered molecular arrangement. On the other hand, it is also related to the higher vibrational coupling associated with molecular rigidity due to molecular connectivity in solution-based measurements. In addition to this, the shape of the absorption spectrum of PTVDPP-BSz changed somewhat from solution to film state, with a distinct shoulder peak appearing near 850 nm. The absorption cut-off value of the polymer at 989 nm results in a bandgap of merely 1.25 eV, implying that it is a well-conducted semiconducting polymer with a narrow optical bandgap. On the other hand, we carried out absorption characterization of monomer TVDPP, and the results are shown in Figure S12. The absorption properties of small molecule are significantly different from those of polymer, which show a triple absorption band. The λ_max_ of TVDPP in the solution and film states are near 656 nm and 677 nm, respectively, which is related to its shorter conjugation length compared to the polymer (Figure S12).

### 3.4. Electrochemical Properties

**Figure 5:**
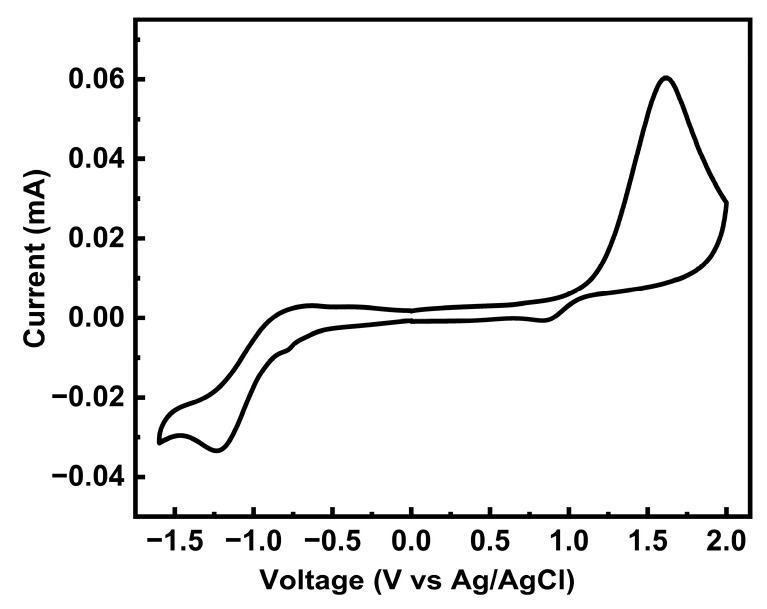
CV patterns of polymer PTVDPP-BSz film (positive swept at 0.1 V/s).

To further characterize the oxidation and reduction behavior and the FMO levels of PTVDPP-BSz, cyclic voltammetry (CV) was employed. The polymer displays a quasi-reversible reduction cycle and an irreversible oxidation profile as shown in Figure 5. According to the onset voltages of its oxidation and reduction peaks, the HOMO and LUMO levels of the material were deduced as −5.62 eV and −3.56 eV, respectively. Thus, the energy level gap of PTVDPP-BSz obtained on the basis of electrochemical tests is 2.06 eV. The difference in the energy level data based on electrochemical and photochemical tests is quite large, which is due to the different testing methods and is common in many of the reported papers [47,48]. PTVDPP-BSz has a high LUMO level and an appropriate HOMO level and therefore has the potential to be a hole-transporting P-type material rather than an electron-transporting material. Meanwhile, the shallow HOMO energy level favors hole injection and transport [2]. The discrepancy between the experimentally derived energy levels of the frontier orbitals and those derived from theoretical simulations is mainly due to the fact that the effective conjugation lengths of the dimers differ significantly from those of the actual macromolecules.

### 3.5. PFET Device Performance

**Figure 6:**
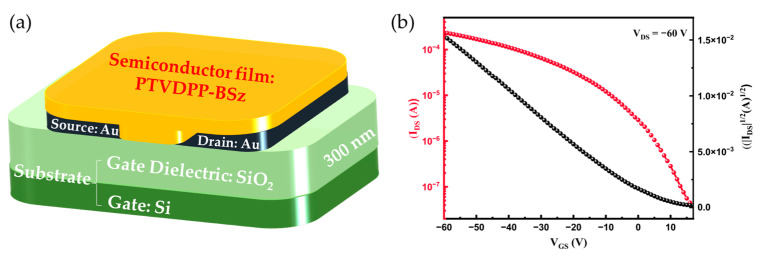
(**a**) PFET-device architecture. (**b**) Representative transfer characteristic curve based on PTVDPP-BSz device.

To evaluate the charge carrier transport properties of the PTVDPP-BSz film, we fabricated a PFET device of BG/BC architecture, as illustrated in Figure 6a. The protocols of device fabrication and characterizations are described in the Materials and Methods section, and the corresponding representative transfer and output characteristic curves are displayed in Figure 6b and Figure S13. We apply a voltage of −60 V between the source and drain electrodes, and the current gradually increases with the increase in the gate voltage, which is typical of p-type materials. The carrier mobilities are extracted from the transfer characteristic curves and calculated by the slope between −10 V and −60 V of the corresponding devices in the saturation regimes as shown in Table 2. The devices achieve a maximum hole mobility (µ_max_) of 0.34 cm^2^ V^−1^ s^−1^ and an average hole mobility (µ_ave_) of 0.32 cm^2^ V^−1^ s^−1^. Moreover, the threshold voltage and current on/off ratio of the device are 4.9 V and 7.63 × 10^3^, respectively. These results show the great potential of materials in the field of PFET devices.

### 3.6. Microstructure and Morphological Analysis of PTVDPP-BSz Film

**Figure 7:**
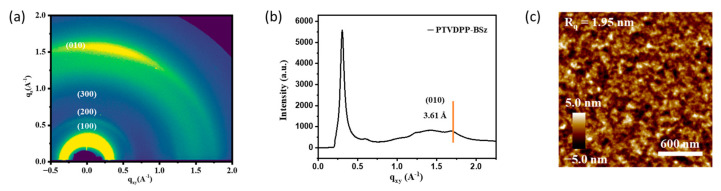
(**a**) 2D-GIWAXS pattern; (**b**) 1D-GIWAXS profile; (**c**) AFM height image of annealed film of PTVDPP-BSz.

The microstructure of PTVDPP-BSz in annealed films was investigated by 2D-GIWAXS. As shown in Figure 7a, the high-order (h00) diffraction peak along the out-of-plane (q_z_) direction has appeared. The polymers show strong (100) and (200) and distinct (300) diffraction peaks, which indicate that they exhibit higher crystallinity than the polymer PDPP-BSz [49]. The pronounced (010) diffraction peaks indicate that the material adopts a face-on stacking mode, which facilitates intermolecular charge hopping through strong π–π stacking [50]. The π–π stacking and d-spacing distance of the polymer were calculated to be 3.61 Å and 19.27 Å, respectively, based on the Bragg equation (Figure 7b and Figure S14). The material shows a tighter stacking distance compared to polymers based on DPP and BSz [51]. The thin film morphology of PTVDPP-BSz was observed using AFM. As shown in Figure 7c, the annealed films show fiber-like networks with a root-mean-square (R_q_) roughness of 1.95 nm. In combination with GIWAXS, the polymer annealed films reveal good crystallinity without significant phase separation, which contributes to highly conductive hole migration.

## 4. Conclusions

In this study, we explored the synthetic route of TVDPP, a new acceptor structural unit distinguished from the classical monomeric TDPP, and obtained the macromolecular material using Suzuki coupling polymerization. The green preparation of the material was contributed by the use of chlorine-free reagents and tin-free reagents. The purified polymers demonstrated good thermal stability and large molecular weight. The material shows a charge transport up to 0.34 cm^2^ V^−1^ s^−1^ in organic transistors and is a potential material with high conductivity for optoelectronic applications. Our laboratory is expanding the preparation of more polymers with this monomer as the core building block to enrich this molecular library.

## Figures and Tables

**Table 1:** The molecular mass of PTVDPP-BSz obtained by high-temperature GPC and its elemental percentage analysis.

	Mn	Mw	Đ ^2^	C ^3^	H ^3^	N ^3^
	(kDa)	(kDa)		(%)	(%)	(%)
PTVDPP-BSz	22.82	49.43	2.16	73.29	8.56	5.67
repeating unit ^1^	-	-	-	73.51	8.87	5.36

^1^ The chemical formula of the min-repeating unit is C_64_H_92_N_4_O_2_S_3_. ^2^ Đ = Mw/Mn. ^3^ Average of results from two separate tests.

**Table 2:** Data on device performance of PTVDPP-BSz.

Coating Speed (rpm)	Annealing Temperature (°C)	µ_max_ ^1^ (cm^2^/(V s))	µ_ave_ ^2^ (cm^2^/(V s))	Threshold Voltage (V)	On/Off Ratio
3000	150	0.34	0.32	4.90	7.63 × 10^3^

^1^ Measured value at saturation regime. ^2^ Average of 10 devices tested.

## Data Availability

All data are included within the article and Supplementary Materials.

## Supplementary Materials

The following supporting information can be downloaded at https://www.mdpi.com/article/10.3390/polym16030396/s1, Figure S1: ^1^H NMR spectrum for compound B; Figure S2: ^13^C NMR spectrum for compound B; Figure S3: ^1^H NMR spectrum for compound C; Figure S4: ^13^C NMR spectrum for compound C; Figure S5: ^1^H NMR spectrum for compound D; Figure S6: ^13^C NMR spectrum for compound D; Figure S7: ^1^H NMR spectrum for TVDPP; Figure S8: ^13^C NMR spectrum for TVDPP; Figure S9: GPC for the polymer; Figure S10: TGA curve of the polymer PTVDPP-BSz (left) and monomer TVDPP (right); Figure S11: Infrared profiles of TVDPP (blue) and PTVDPP-BSz (red); Figure S12: UV-vis absorption curve of TVDPP; Figure S13: The output curves based on the PTVDPP-BSz device; Figure S14: 1D-GIWAXS profile of annealed film of PTVDPP-BSz.

## References

[1] Zhang Q., Hu W., Sirringhaus H., Müllen K. (2022). Recent Progress in Emerging Organic Semiconductors. Adv. Mater..

[2] Zhang S., Li W., Chen Y., Wu Z., Chen Z., Zhao Y., Wang Y., Liu Y. (2023). Perylenediimide regioisomers with tunable physicochemical and charge-transport properties. Chem. Commun..

[3] Holliday S., Donaghey J.E., McCulloch I. (2013). Advances in Charge Carrier Mobilities of Semiconducting Polymers Used in Organic Transistors. Chem. Mater..

[4] Wang Y., Liu Y. (2023). Insight into conjugated polymers for organic electrochemical transistors. Trends Chem..

[5] Yang J., Zhao Z., Wang S., Guo Y., Liu Y. (2018). Insight into High-Performance Conjugated Polymers for Organic Field-Effect Transistors. Chem.

[6] Głowacki E.D., Voss G., Sariciftci N.S. (2013). 25th Anniversary Article: Progress in Chemistry and Applications of Functional Indigos for Organic Electronics. Adv. Mater..

[7] Dai C., Liu Y., Wei D. (2022). Two-Dimensional Field-Effect Transistor Sensors: The Road toward Commercialization. Chem. Rev..

[8] Cao X., Han Y. (2024). Control over the aggregated structure of donor–acceptor conjugated polymer films for high-mobility organic field-effect transistors. Aggregate.

[9] Xu X., Zhao Y., Liu Y. (2023). Wearable Electronics Based on Stretchable Organic Semiconductors. Small.

[10] Ren S., Habibi A., Ni P., Nahdi H., Bouanis F.Z., Bourcier S., Clavier G., Frigoli M., Yassar A. (2023). Synthesis and characterization of solution-processed indophenine derivatives for function as a hole transport layer for perovskite solar cells. Dye. Pigment..

[11] Bronstein H., Nielsen C.B., Schroeder B.C., McCulloch I. (2020). The role of chemical design in the performance of organic semiconductors. Nat. Rev. Chem..

[12] Sun H., Guo X., Facchetti A. (2020). High-Performance n-Type Polymer Semiconductors: Applications, Recent Development, and Challenges. Chem.

[13] Zhu M., Guo Y., Liu Y. (2022). A thriving decade: Rational design, green synthesis, and cutting-edge applications of isoindigo-based conjugated polymers in organic field-effect transistors. Sci. China Chem..

[14] Ko E.Y., Park G.E., Lee D.H., Um H.A., Shin J., Cho M.J., Choi D.H. (2015). Enhanced Performance of Polymer Solar Cells Comprising Diketopyrrolopyrrole-Based Regular Terpolymer Bearing Two Different π-Extended Donor Units. ACS Appl. Mater. Interfaces.

[15] Zhao Y., Guo Y., Liu Y. (2013). 25th Anniversary Article: Recent Advances in n-Type and Ambipolar Organic Field-Effect Transistors. Adv. Mater..

[16] Ren S., Habibi A., Ni P., Zhang Y., Yassar A. (2023). Tuning the Photophysical Properties of Acceptor–Donor–Acceptor Di-2-(2-oxindolin-3-ylidene) Malononitrile Materials via Extended π–Conjugation: A Joint Experimental and Theoretical Study. Materials.

[17] Ding L., Yu Z.-D., Wang X.-Y., Yao Z.F., Lu Y., Yang C.-Y., Wang J.Y., Pei J. (2023). Polymer Semiconductors: Synthesis, Processing, and Applications. Chem. Rev..

[18] Zhou Y., Zhang W., Yu G. (2021). Recent structural evolution of lactam- and imide-functionalized polymers applied in organic field-effect transistors and organic solar cells. Chem. Sci..

[19] Li Y., Sonar P., Murphy L., Hong W. (2013). High mobility diketopyrrolopyrrole (DPP)-based organic semiconductor materials for organic thin film transistors and photovoltaics. Energy Environ. Sci..

[20] Liu Q., Bottle S.E., Sonar P. (2019). Developments of Diketopyrrolopyrrole-Dye-Based Organic Semiconductors for a Wide Range of Applications in Electronics. Adv. Mater..

[21] Shi L., Guo Y., Hu W., Liu Y. (2017). Design and effective synthesis methods for high-performance polymer semiconductors in organic field-effect transistors. Mater. Chem. Front..

[22] Carsten B., He F., Son H.J., Xu T., Yu L. (2011). Stille Polycondensation for Synthesis of Functional Materials. Chem. Rev..

[23] Mueller C.J., Singh C.R., Fried M., Huettner S., Thelakkat M. (2015). High Bulk Electron Mobility Diketopyrrolopyrrole Copolymers with Perfluorothiophene. Adv. Funct. Mater..

[24] Li Y., Sonar P., Singh S.P., Soh M.S., van Meurs M., Tan J. (2011). Annealing-Free High-Mobility Diketopyrrolopyrrole−Quaterthiophene Copolymer for Solution-Processed Organic Thin Film Transistors. J. Am. Chem. Soc..

[25] Li Y., Singh S.P., Sonar P. (2010). A High Mobility P-Type DPP-Thieno[3,2-b]thiophene Copolymer for Organic Thin-Film Transistors. Adv. Mater..

[26] Hong J., Kim J., Li Z., Cong C., Rand B.P., Nam S.Y., Kim S.H., Kim Y.H. (2023). Facile Direct Printing of DPP-Based Polymers for Organic Field-Effect Transistors and Logic Gates. ACS Appl. Electron. Mater..

[27] Kim H.S., Huseynova G., Noh Y.-Y., Hwang D.-H. (2017). Modulation of Majority Charge Carrier from Hole to Electron by Incorporation of Cyano Groups in Diketopyrrolopyrrole-Based Polymers. Macromolecules.

[28] Nielsen C.B., Turbiez M., McCulloch I. (2012). Recent Advances in the Development of Semiconducting DPP-Containing Polymers for Transistor Applications. Adv. Mater..

[29] Shen T., Li W., Zhao Y., Wang Y., Liu Y. (2022). A Hybrid Acceptor-Modulation Strategy: Fluorinated Triple-Acceptor Architecture for Significant Enhancement of Electron Transport in High-Performance Unipolar n-Type Organic Transistors. Adv. Mater..

[30] Pop F., Lewis W., Amabilino D.B. (2016). Solid state supramolecular structure of diketopyrrolopyrrole chromophores: Correlating stacking geometry with visible light absorption. CrystEngComm.

[31] Anthony J.E., Facchetti A., Heeney M., Marder S.R., Zhan X. (2010). n-Type Organic Semiconductors in Organic Electronics. Adv. Mater..

[32] Humphreys J., Pop F., Hume P.A., Murphy A.S., Lewis W., Davies E.S., Argent S.P., Amabilino D.B. (2021). Solid state structure and properties of phenyl diketopyrrolopyrrole derivatives. CrystEngComm.

[33] Zou X., Cui S., Li J., Wei X., Zheng M. (2021). Diketopyrrolopyrrole Based Organic Semiconductor Materials for Field-Effect Transistors. Front. Chem..

[34] Li W., Lee T., Oh S.J., Kagan C.R. (2011). Diketopyrrolopyrrole-Based π-Bridged Donor–Acceptor Polymer for Photovoltaic Applications. ACS Appl. Mater. Interfaces.

[35] Zhang W., Shi K., Lai J., Zhou Y., Wei X., Che Q., Wei J., Wang L., Yu G. (2023). Record-High Electron Mobility Exceeding 16 cm2 V^−1^ s^−1^ in Bisisoindigo-Based Polymer Semiconductor with a Fully Locked Conjugated Backbone. Adv. Mater..

[36] Shen T., Li W., Zhao Y., Liu Y., Wang Y. (2022). An all-C–H-activation strategy to rapidly synthesize high-mobility well-balanced ambipolar semiconducting polymers. Matter.

[37] Ran Y., Guo Y., Liu Y. (2020). Organostannane-free polycondensation and eco-friendly processing strategy for the design of semiconducting polymers in transistors. Mater. Horiz..

[38] Wang Y., Michinobu T. (2016). Benzothiadiazole and its π-extended, heteroannulated derivatives: Useful acceptor building blocks for high-performance donor–acceptor polymers in organic electronics. J. Mater. Chem. C.

[39] Chen Y., Wu Z., Ding L., Zhang S., Chen Z., Li W., Zhao Y., Wang Y., Liu Y. (2023). Manipulating Crystal Stacking by Sidechain Engineering for High-Performance N-Type Organic Semiconductors. Adv. Funct. Mater..

[40] Chen L., Qin Z., Huang H., Zhang J., Yin Z., Yu X., Zhang X.s., Li C., Zhang G., Huang M. (2023). High-Performance Ambipolar and n-Type Emissive Semiconductors Based on Perfluorophenyl-Substituted Perylene and Anthracene. Adv. Sci..

[41] Shi Y., Li W., Wang X., Tu L., Li M., Zhao Y., Wang Y., Liu Y. (2022). Isomeric Acceptor–Acceptor Polymers: Enabling Electron Transport with Strikingly Different Semiconducting Properties in n-Channel Organic Thin-Film Transistors. Chem. Mater..

[42] Wang Y., Kadoya T., Wang L., Hayakawa T., Tokita M., Mori T., Michinobu T. (2015). Benzobisthiadiazole-based conjugated donor–acceptor polymers for organic thin film transistors: Effects of π-conjugated bridges on ambipolar transport. J. Mater. Chem. C.

[43] Luo X., Shen H., Perera K., Tran D.T., Boudouris B.W., Mei J. (2021). Designing Donor–Acceptor Copolymers for Stable and High-Performance Organic Electrochemical Transistors. ACS Macro Lett..

[44] Frisch M.J., Trucks G., Schlegel H.B., Scuseria G.E., Robb M.A., Cheeseman J.R., Scalmani G., Barone V., Mennucci B., Petersson G.A. (2009). Gaussian 09W, revision A. 02..

[45] Lee C., Yang W., Parr R.G. (1988). Development of the Colle-Salvetti correlation-energy formula into a functional of the electron density. Phys. Rev. B Condens. Matter.

[46] Zhao Y., Li W., Shen T., Zhao Y., Liu Y., Wang Y. (2022). The marriage of dual-acceptor strategy and C-H activation polymerization: Naphthalene diimide-based n-type polymers with adjustable molar mass and decent performance. Sci. China Chem..

[47] Jeong W., Lee K., Jang J., Jung I.H. (2023). Development of Benzobisoxazole-Based Novel Conjugated Polymers for Organic Thin-Film Transistors. Polymers.

[48] Cho S., Lee J., Tong M., Seo J.H., Yang C. (2011). Poly(diketopyrrolopyrrole-benzothiadiazole) with Ambipolarity Approaching 100% Equivalency. Adv. Funct. Mater..

[49] Gruber M., Jung S.-H., Schott S., Venkateshvaran D., Kronemeijer A.J., Andreasen J.W., McNeill C.R., Wong W.W.H., Shahid M., Heeney M. (2015). Enabling high-mobility, ambipolar charge-transport in a DPP-benzotriazole copolymer by side-chain engineering. Chem. Sci..

[50] Yang J., Wang H., Chen J., Huang J., Jiang Y., Zhang J., Shi L., Sun Y., Wei Z., Yu G. (2017). Bis-Diketopyrrolopyrrole Moiety as a Promising Building Block to Enable Balanced Ambipolar Polymers for Flexible Transistors. Adv. Mater..

[51] Kim J., Han A.R., Hong J., Kim G., Lee J., Shin T.J., Oh J.H., Yang C. (2014). Ambipolar Semiconducting Polymers with π-Spacer Linked Bis-Benzothiadiazole Blocks as Strong Accepting Units. Chem. Mater..

