# Supplementary material for: Design of Novel Functional Conductive Structures and Preparation of High-Hole-Mobility Polymer Transistors by Green Synthesis Using Acceptor–Donor–Acceptor Strategies
Source: Polymers (Basel). 2024 Jan 31;16(3):396. doi: 10.3390/polym16030396 (PMC10857718; doi:10.3390/polym16030396)

Supplementary Materials

Figure S1. ^{1}H NMR spectrum for compound B.

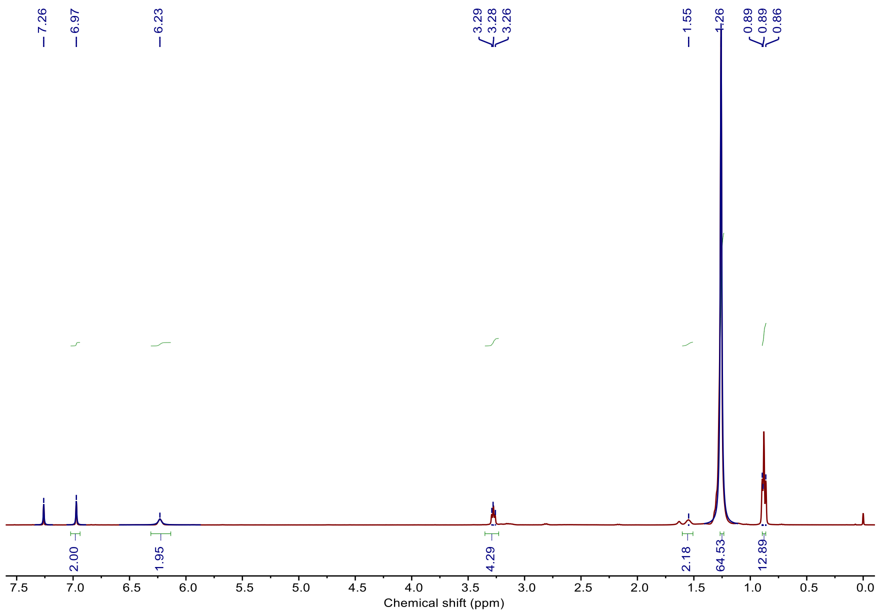

^{1}H NMR (400 MHz, $CDCl_3$, ppm): 6.97 (s, 2H), 6.23 (br, 2H), 3.28 (t, J = 5.9 Hz, 4H), 1.60−1.49 (m, 2H), 1.36−1.11 (m, 64H), 0.89 (t, J = 6.7 Hz, 12H).

Figure S2. ^{13}C NMR spectrum for compound B.

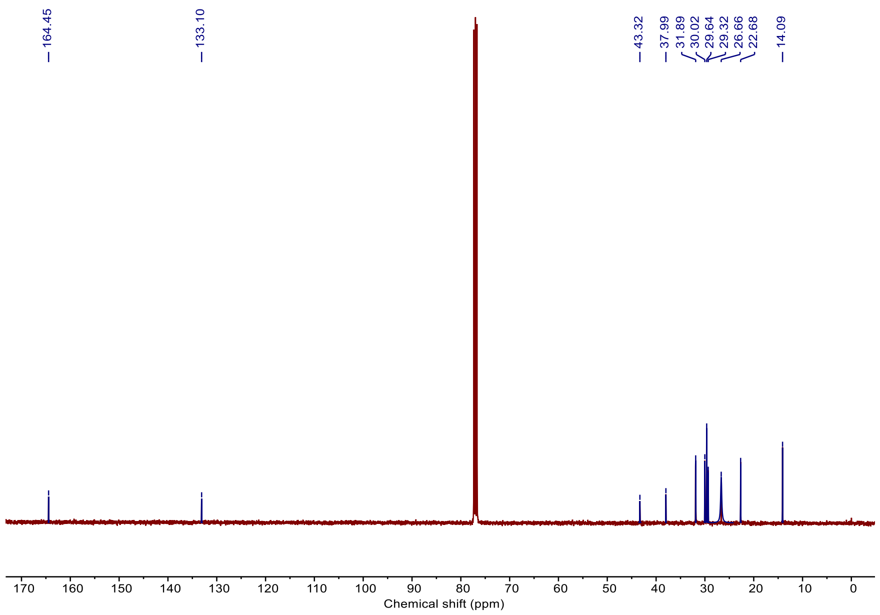

^{13}C NMR (100 MHz, $CDCl_3$, ppm): 164.45, 133.10, 43.32, 37.99, 31.89, 30.02, 29.64, 29.32, 26.66, 22.68, 14.09.

Figure S3. ^{1}H NMR spectrum for compound C.

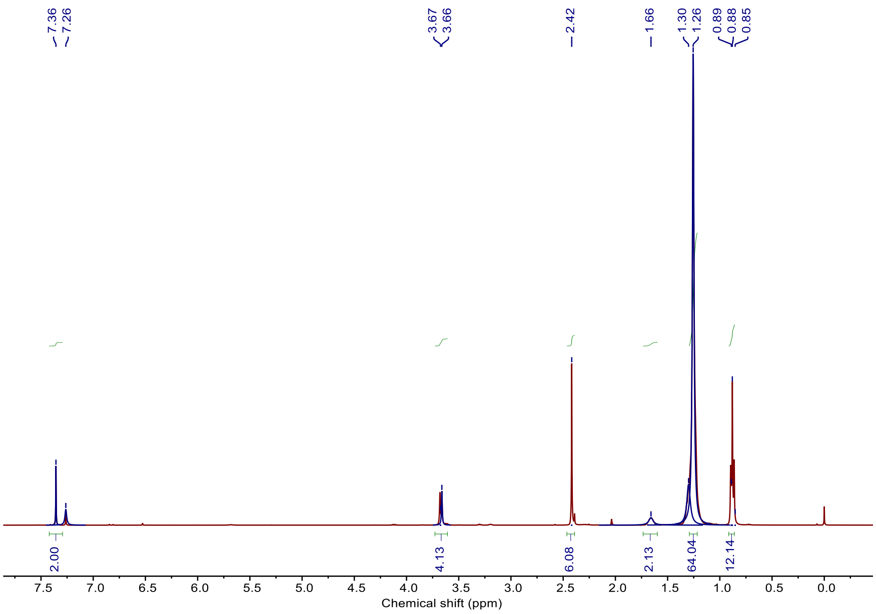

^{1}H NMR (400 MHz, $CDCl_3$, ppm): 7.36 (s, 2H), 3.66 (d, J = 7.5 Hz, 4H), 2.42 (s, 6H), 1.70−1.60 (m, 2H), 1.40−1.20 (m, 64H), 0.88 (t, J = 6.8 Hz, 12H).

Figure S4. ^{13}C NMR spectrum for compound C.

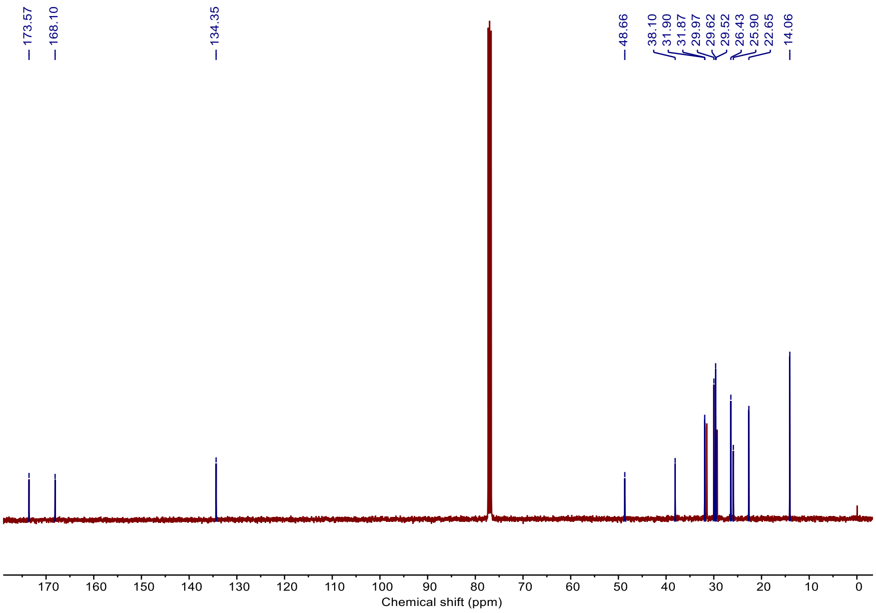

^{13}C NMR (100 MHz, $CDCl_3$, ppm): 173.57, 168.10, 134.35, 48.66, 38.10, 31.90, 31.87, 29.97, 29.62, 29.52, 26.43, 25.90, 22.65, 14.06.

Figure S5. ^{1}H NMR spectrum for compound D.

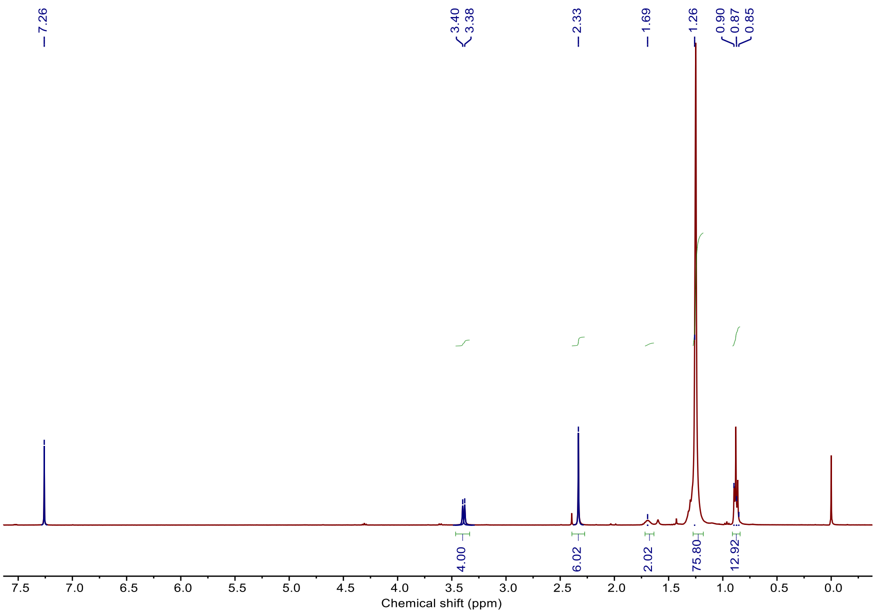

^{1}H NMR (400 MHz, $CDCl_3$, ppm): 3.40 (d, J = 7.6 Hz, 4H), 2.33 (s, 6H), 1.74−1.59 (m, 2H), 1.37−1.19 (s, 64H), 0.87 (t, J = 6.9 Hz, 12H).

Figure S6. ^{13}C NMR spectrum for compound D.

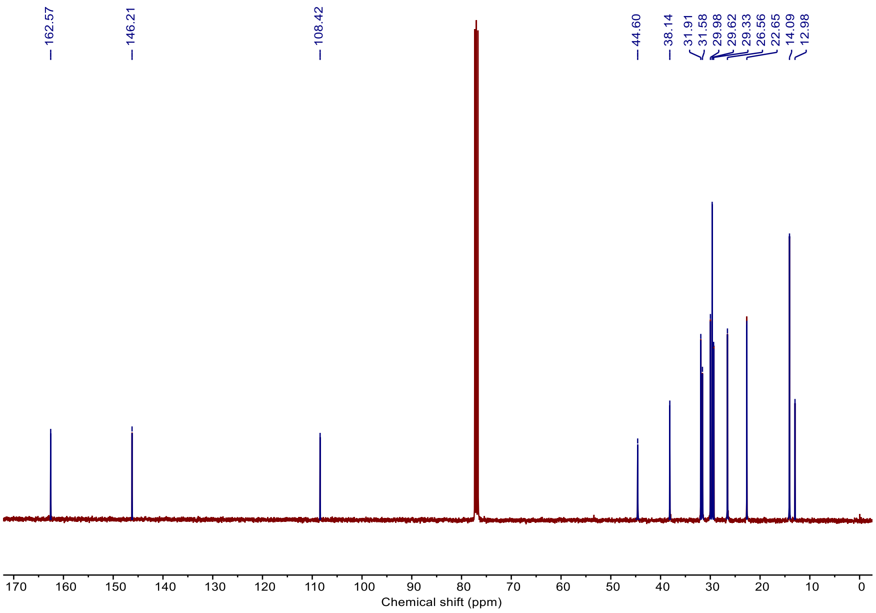

^{13}C NMR (100 MHz, $CDCl_3$, ppm): 162.57, 146.21, 108.42, 44.60, 38.14, 31.91, 31.58, 29.98, 29.62, 29.33, 26.56, 22.65, 14.09, 12.98.

Figure S7. ^{1}H NMR spectrum for TVDPP.

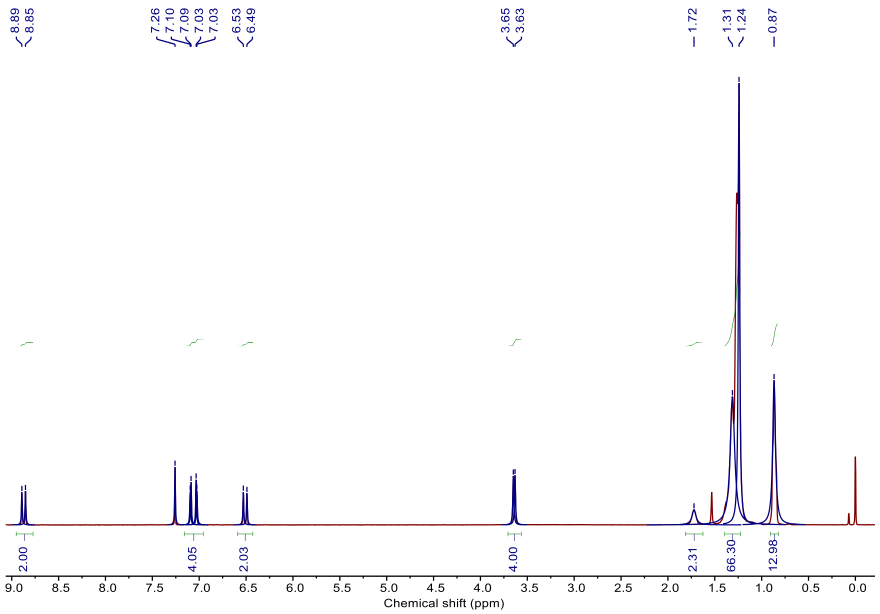

^{1}H NMR (400 MHz, $CDCl_3$, ppm): 8.87 (d, J = 15.3 Hz, 2H), 7.10−7.03 (m, 4H), 6.51 (d, J = 15.4 Hz, 2H), 3.64 (d, J = 7.3 Hz, 4H), 1.78−1.65 (m, 2H), 1.38−1.20 (m, 64H), 0.87 (t, J = 6.9 Hz, 12H).

Figure S8. ^{13}C NMR spectrum for TVDPP.

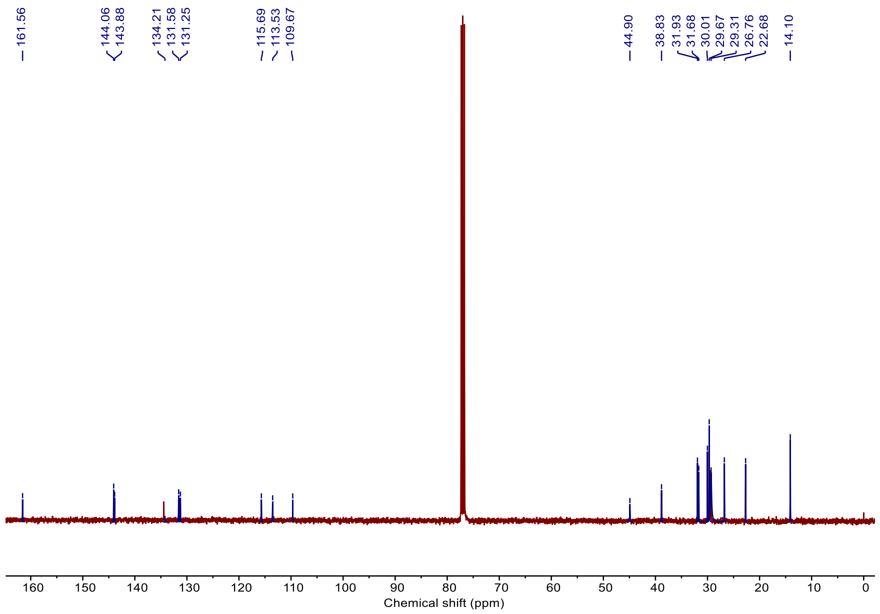

^{13}C NMR (100 MHz, $CDCl_3$, ppm): 161.56, 144.06, 143.88, 134.21, 131.58, 131.25, 115.69, 113.53, 109.67, 44.90, 38.83, 31.93, 31.68, 30.01, 29.67, 26.76, 22.68, 14.10.

Figure S9. GPC for the polymer.

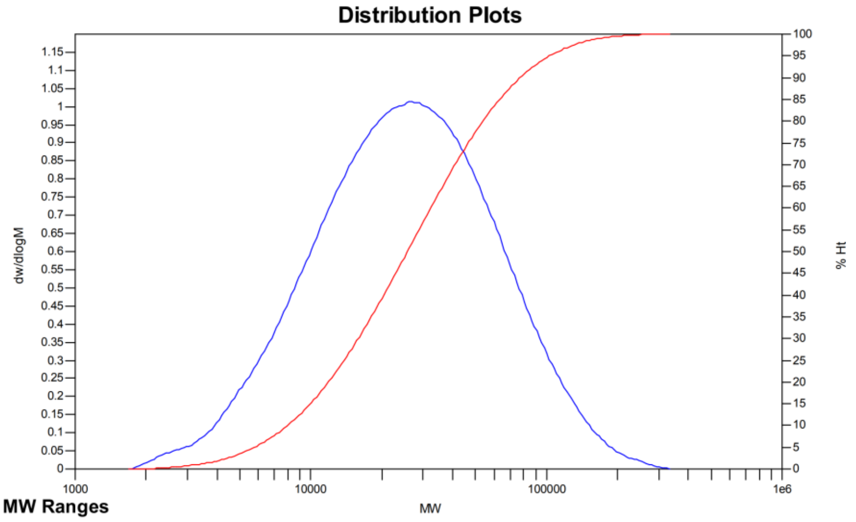

Figure S10. TGA curve of the polymer PTVDPP-BSz (left) and monomer TVDPP (right).

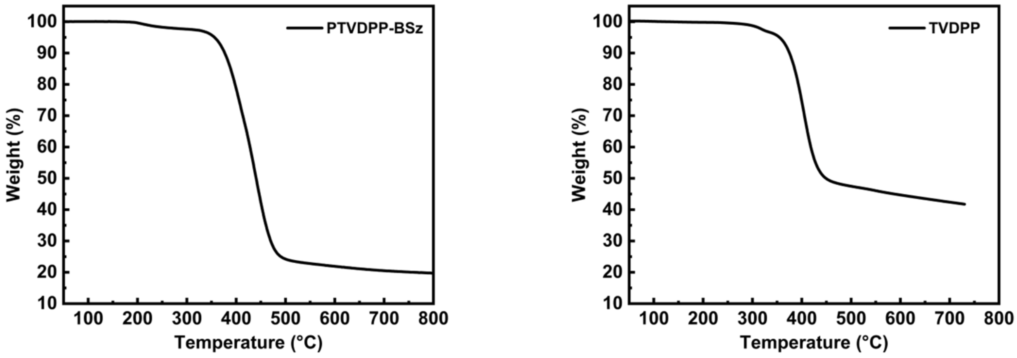

Figure S11: Infrared profiles of TVDPP (blue) and PTVDPP-BSz (red)

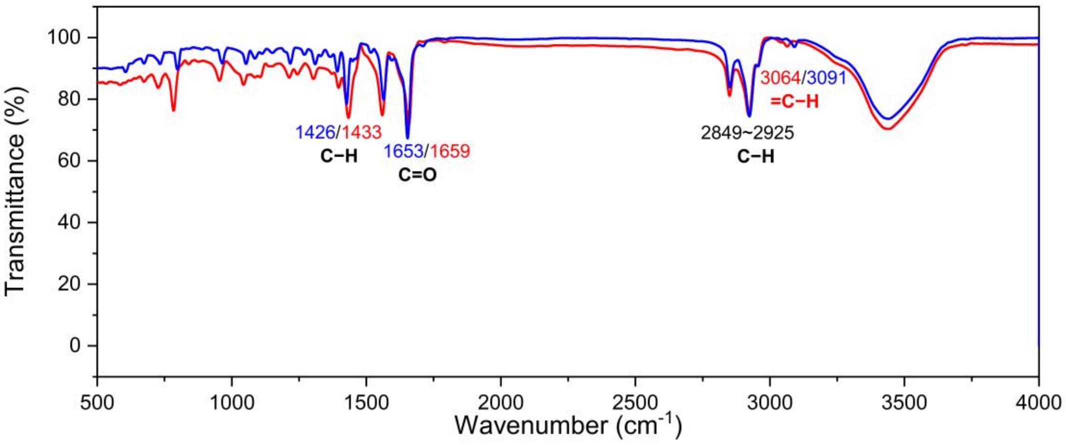

Figure S12: UV-vis absorption curve of TVDPP

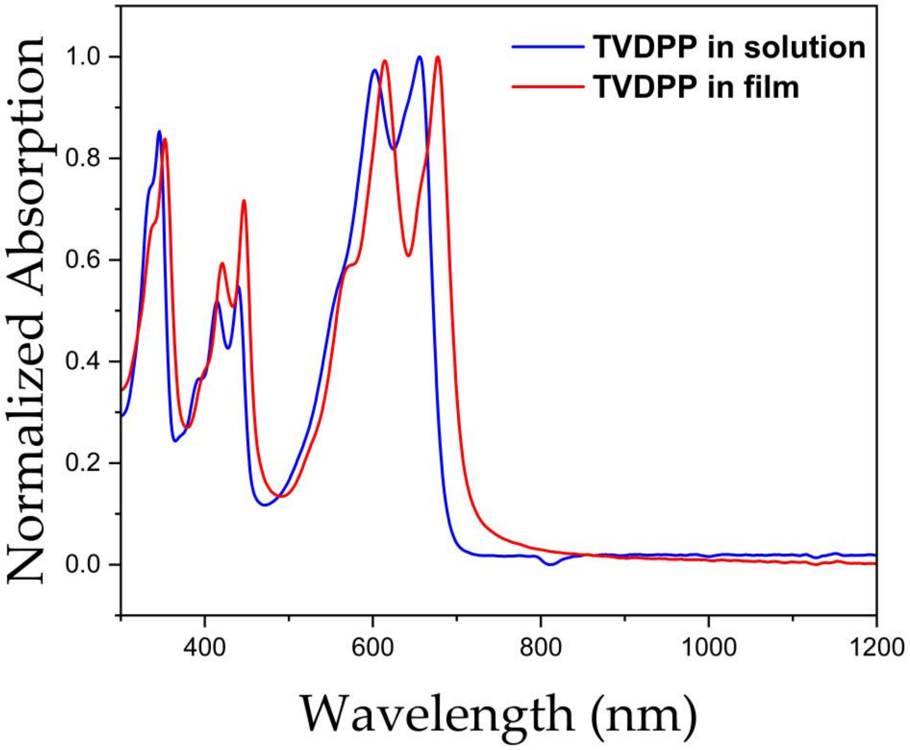

Figure S13. The output curves based on the PTVDPP-BSz device

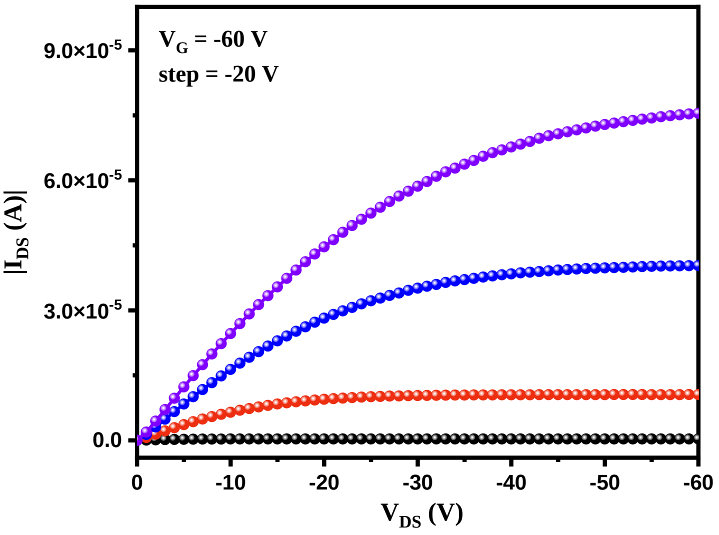

Figure S14. 1D-GIWAXS profile of annealed film of PTVDPP-BSz.

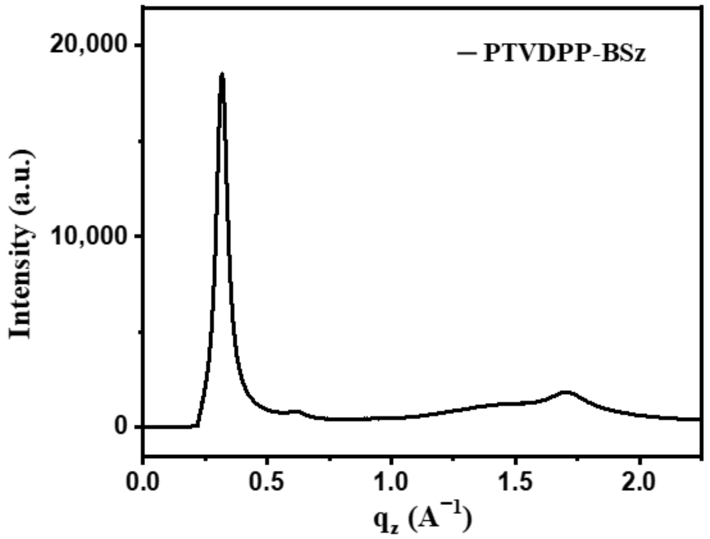

Supplement: Supplementary file 1 [file polymers-16-00396-s001.zip › polymers-2836733-supplementary.pdf]
